# Exploring the inflammatory origins of gouty arthritis: mechanistic studies based on local lesion proteomics and validation

**DOI:** 10.3389/fimmu.2026.1764497

**Published:** 2026-03-25

**Authors:** Niqin Xiao, Hongting Lu, Sanjin Zeng, Heguo Yan, Qianqian Yang, Lihe Sheng, Bingbing Chen, Yundong Xu, Jian Zhang, Jing Xie, Weijian Zhou, Zhaofu Li, Zhaohu Xie

**Affiliations:** 1 Yunnan University of Chinese Medicine, Kunming, China; 2 Joint Graduate School of Traditional Chinese Medicine of China, Suzhou, China; 3 The First Affiliated Hospital of Yunnan University of Chinese Medicine, Kunming, China

**Keywords:** gouty arthritis, disease staging, proteomics, validation, joint cavity effusion, tophi, ferroptosis

## Abstract

**Background:**

The exact pathogenesis of gouty arthritis (GA) is unclear. However, it is believed that GA is characterized by recurrent episodes and a self-limiting nature. During episodes, the disease primarily manifests locally, making local omics research particularly relevant.

**Objective:**

Based on the local proteomic analysis of GA patients during the chronic phase (with tophi) and acute phase (with joint cavity effusion), this study systematically screened for inflammation-related differentially expressed proteins to provide specific biomarkers and potential intervention targets for disease prevention and treatment.

**Methods:**

Through a cross-sectional study, the baseline data were collected from the GA patients with chronic phase (with tophi) and acute phase (with joint cavity effusion)(n = 100 per group). Five cases were randomly selected from each group for local proteomics analysis to determine the biological functions and enriched signaling pathways of differentially expressed proteins. Based on the differential results, 30 additional cases were randomly selected from each group for validation using Western blot analysis.

**Results:**

The quantitative proteomics analysis identified a total of 810 differentially expressed proteins between the two groups, including 548 upregulated and 262 downregulated proteins. The GO and KEGG pathway enrichment analysis showed that the differential proteins were associated with ferroptosis. Ultimately, three ferroptosis-related differential proteins, including glutathione peroxidase 4 (GPX4), ferritin heavy chain 1 (FTH1), arachidonate and 15-lipoxygenase (ALOX15) were identified in GA patients. The differential expression of these proteins was validated using Western blot, showing statistically significant differences (*P* < 0.05).

**Conclusions:**

The comprehensive analysis of proteomics and Western blot validation experiments showed that local lesions in the chronic phase of GA exhibited increased expression levels of GPX4 and FTH1, while local lesions in the acute phase of GA exhibited increased expression of ALOX15. Therefore, it was speculated that ferroptosis-inhibiting factors in the chronic phase of GA might participate in the chronic progression of the disease by limiting the toxicity of free iron. In the acute phase of GA, ferroptosis-promoting factors were activated in the joint microenvironment. This study revealed the differential expression of ferroptosis-related proteins at different stages of GA, providing a theoretical basis for developing GA staging and treatment strategies based on ferroptosis regulation.

## 1 Introduction

Currently, gouty arthritis (GA) is one of the most common inflammatory joint diseases (1), as well as the most typical clinical manifestation of gout. It has a complex and poorly understood pathogenesis mechanism. However, it is generally believed to be a metabolic disorder in which serum uric acid (SUA) levels exceed the saturation point in blood or tissue fluid. This leads to the formation of monosodium urate (MSU) crystals that deposit in joints, triggering inflammatory reactions and tissue damage. Epidemiological studies have shown that the global prevalence of gout currently ranges from 1-6.8% (2), with an increasing trend over the years. Its incidence rate in males is 3 times higher than in females (3).

**Figure 1:**
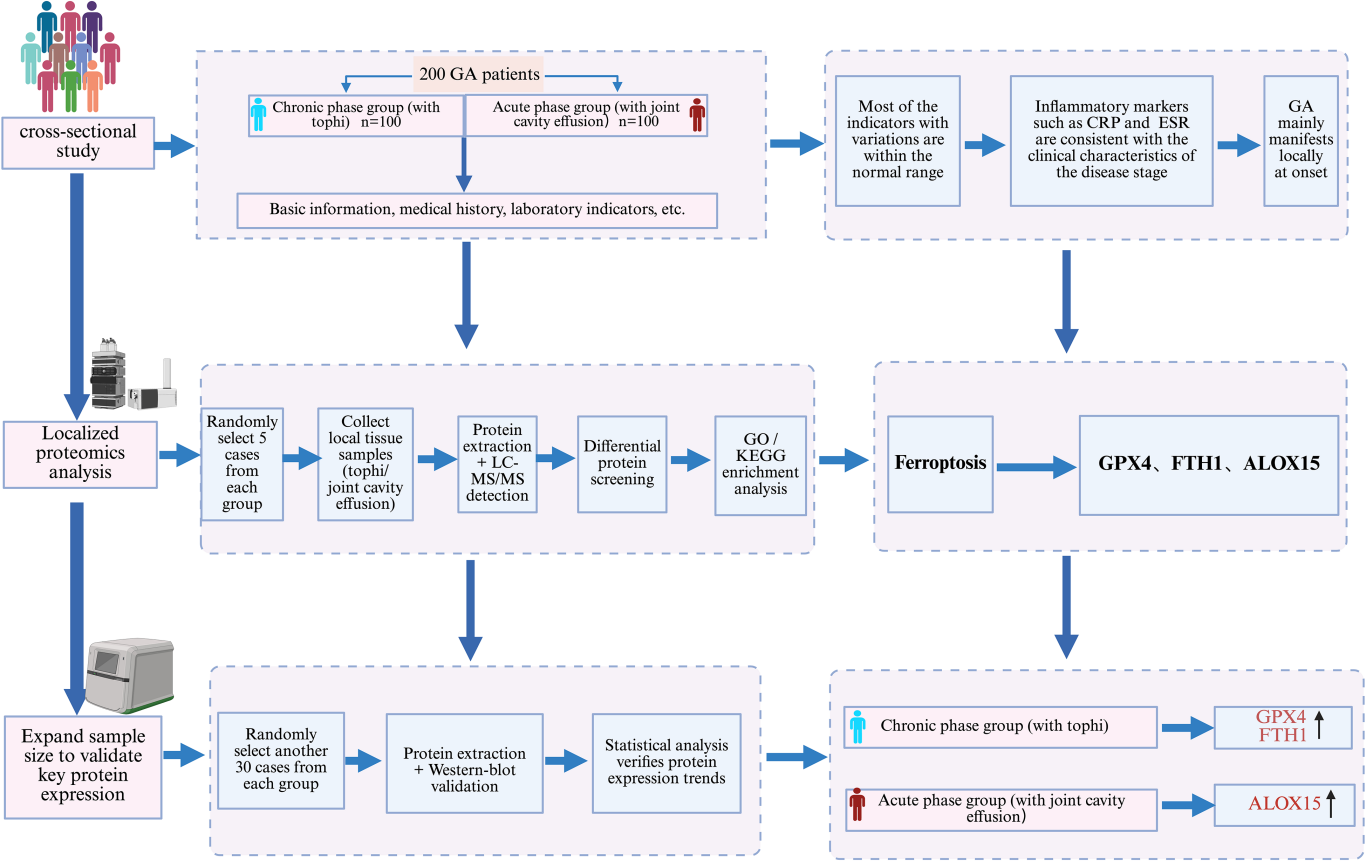
Research approach diagram.

Previous studies (4–6) have shown that the pathogenesis of GA is primarily caused by the persistent hyperuricemia and/or the activation of the innate immune system by MSU crystals deposited in joints and other sites, leading to inflammatory responses. Under the influence of chemokines, a large number of immune cells migrate to the affected area. In gout, MSU acts as a Damage Associated Molecular Patterns(DAMPs), serving as a danger signal, activating multiple immune cells (7). This might involve a crosstalk during the clearance of MSU, leading to programmed cell death and releasing a large number of inflammatory factors, thereby maintaining or exacerbating the inflammatory response. However, the exact pathogenesis of GA remains unclear. It is characterized by recurrent episodes and a self-limiting nature, with predominant local manifestations during episodes. Therefore, localized omics studies are highly necessary. Proteomics analysis compares protein expression profiles under different physiological or pathological conditions, thereby identifying differentially expressed proteins. These differentially expressed proteins might serve as disease biomarkers, drug targets, molecules in key signaling pathways, or key factors in understanding specific biological processes (8–10). The current study aimed to identify inflammatory protein signals in tophi and joint cavity effusion obtained from chronic-phase and acute-phase GA patients, respectively. This study further validated the differences in protein expression levels between the two phases, aiming to provide new biomarkers for clinical diagnosis and guide new targeted therapies (**Figure 1**).

## 2 Research subjects and methods

### 2.1 Baseline data collection

A cross-sectional study was conducted to collect the baseline data from 100 patients with chronic GA complicated by tophi and 100 patients with acute GA complicated by joint cavity effusion. All the patient data was collected from the Department of Rheumatology, the First Affiliated Hospital of Yunnan University of Chinese Medicine.

### 2.2 Research subjects

This study employed a two-stage, non-overlapping sample selection strategy. In the first stage, to perform unbiased differential protein screening, five samples per group were independently selected from the 200 baseline cohort meeting postoperative sampling criteria using a computerized random number table for proteomic analysis. This sample size was conventionally determined based on preliminary exploratory studies, enabling efficient capture of major expression profile characteristics while conserving clinical samples. All samples underwent strict 4 °C cold chain transportation to ensure protein stability. Based on the list of differentially expressed proteins obtained from the omics analysis, we proceeded to the second validation phase. To ensure validation independence, we first excluded the 5 samples used in the omics analysis. Subsequently, 30 new samples were independently and randomly selected from the remaining eligible patients in both groups. Target proteins were validated using Western Blot.

The patient samples used in this study (tophi and joint cavity effusion) are residual specimens obtained from patients following tophi excision and joint cavity effusion aspiration procedures at the hospital, which were no longer required for clinical diagnosis or treatment. All samples were derived from completed routine medical procedures, and no additional interventions were performed on patients during the research process. This sample collection protocol and baseline data acquisition have been reviewed and approved by the Ethics Committee of the First Affiliated Hospital of Yunnan University of Chinese Medicine(No.YYLW-2025-009).

#### 2.2.1 Diagnostic and staging criteria

According to the 2015 gout classification criteria: an American College of Rheumatology/European League Against Rheumatism collaborative initiative (11), a cumulative score of ≥8 points was diagnosed as gout. In the acute phase, the initial symptom was an acute gout attack, characterized by sudden joint redness, swelling, and pain, often affecting one side. During the intercritical phase, there were typically no symptoms or only mild joint symptoms, and the diagnosis relied on the history of acute GA and hyperuricemia. In the chronic phase, the disease had a long course, with the formation of tophi, which was associated with poor control of hyperuricemia.

#### 2.2.2 Inclusion criteria

Eligible patients must be between 18 and 70 years of age, with no restriction on gender (**Table 1**).

**Table 1:** Inclusion criteria.

Conditions	Specific explanation
Age	18–70 years old
Gender	unlimited
Others The patients with tophi in the chronic phase, without joint pain, underwent tophi removal due to concerns about aesthetic appearance or mobility. The patients with joint cavity effusion did not receive anti-inflammatory analgesic treatment within 48 hours, such as etoricoxib or colchicine.	

#### 2.2.3 Exclusion criteria

The exclusion criteria for the recruitment of patients were as follows :(1) secondary gout ;(2) intolerable joint pain (visual analogue scale >7); (3) elevated liver and kidney function tests, such as ALT, AST, Cr, and BUN levels exceeding 1.5 times the upper limit of normal; (4) pregnant or breastfeeding women; (5) concurrent conditions, such as malignant tumors or gastrointestinal ulcers; (6) currently using medications that interfere with uric acid metabolism, or a history of other medication abuse.

### 2.3 Main materials and instruments used in the experiment

RIGOL L-3000 High Performance Liquid Chromatography System (Beijing Puyuan Jingdian Technology Co., Ltd.) Vortex Mixer (SCILOGEX, Model: MX-S), Vacuum Centrifugal Concentrator (Beijing Ji’ai Mu Technology Co., Ltd., Model: CV100-DNA), Electric Heating Constant Temperature Water Bath (Beijing Guangming Medical Instrument Co., Ltd., Model: XMTD-7000), Centrifuge (Eppendorf), Microplate Reader (DR200B), Electrophoresis System (Bio-Rad), High-Throughput Tissue Grinder (Shanghai Hefan Instruments Co., Ltd., Model: hf-48), Ultrasonic Homogenizer (Shanghai Huxi Industrial Co., Ltd., Model: JY96-IIN), 10K Ultrafiltration Tubes (Sartorius, PN: VN01H02). Chemiluminescence imaging system (Bio-Rad, USA, ChemiDoc).

Ammonium bicarbonate (Sigma-Aldrich, A6141-500G), TEAB (Sigma-Aldrich, T7408-100mL), Urea (Amresco, M123-1KG), Protein Quantification Dye (Huaxingbio, HXJ5137), Bovine Serum Albumin (Thermo Scientific, 23209), Dithiothreitol DTT (Amresco, M109-5G), Iodoacetamide IAM (Amresco, M216-30G), Trypsin (Promega, V5280/100ug), Ziptip (Millipore, ZTC18M096), Acetonitrile (J.T.Baker, 34851 MSDS), Ammonia solution (Wako Pure Chemical Industries Ltd, 013-23355), Formic acid (Sigma-Aldrich, T79708), Sample vials (Thermo, 11190533), Vial caps (Thermo, 11150635). Plasma Low Abundance Enrichment Magnetic Bead Kit QLBIO MagicOmics-DMB8X, Blood Exosome Enrichment Magnetic Bead Kit QLBIO MagicOmics-EMB8X, Micro/Universal Proteomics Kit QLBIO MagicOmics-MMB8X, Proteomics Robot QLBIO MagicOmics-AP-96, TGX Stain-Free FastCast Acrylamide Kit(Bio-Rad, #1610183), Universal Antibody Dilution Buffer (Yamei PS119L), anti-arachidonate 15-lipoxygenase(anti-ALOX15,BBI LIFE SCIENCES CORPORATION D161433), anti-glutathione peroxidase 4(anti-GPX4,Bioswamp PAB47937), ferritin heavy chain 1(anti-FTH1,BBI LIFE SCIENCES CORPORATION D290553), goat anti-rabbit IgG (BBI LIFE SCIENCES CORPORATION D110058), ECL Chemiluminescent Reagent (Millipore WBKLS0500).

### 2.4 Screening of differentially expressed proteins

#### 2.4.1 Enzymatic desalination

The protein samples were prepared for spectrometry analysis using the QLBIO MagicOmics-MMB8X micro/universal proteome digestion kit. Briefly, a 20-μL protein sample was added to the eight-well strip containing MMB beads and incubated at 37 °C for 30 minutes. Then, 45 μL of binding buffer was added and incubated at room temperature with gentle shaking for 15 minutes. After incubation, the supernatant was discarded, followed by washing the MMB beads three times with washing buffer. Then, 20 μL of digestion working solution was added to resuspend the magnetic beads and incubated at 37 °C for over 4 hours. After incubation, 5μL of Quench buffer was added to terminate the digestion, followed by lyophilization.

#### 2.4.2 LC-MS/MS analysis

Mobile phase A (100% water, 0.1% formic acid) and mobile phase B (80% acetonitrile, 0.1% formic acid) were prepared. Resuspend the lyophilized peptide powder obtained after enzymatic digestion and desalting was dissolved in 10 µL of mobile phase A and centrifuged at 14,000 g at 4 °C for 20 minutes. Then, 1µg of the supernatant was injected for liquid chromatography-mass spectrometry (LC-MS) analysis using the Orbitrap Exploris™ 480 mass spectrometer with the FAIMS Pro™ Interface. The compensation voltage (CV) switched between –45 and –65 every 1 second. The Nanospray Flex™ (NSI) ion source was set with an ion spray voltage of 2.0 kV and an ion transfer tube temperature of 320 °C. The mass spectrometer operated in data-dependent acquisition mode, with a full scan range of m/z of 350–1500, primary mass spectrometer resolution of 120,000 (at 200 m/z), Automatic Gain Control(AGC) of 300%, and C-trap maximum injection time of 50 ms. The secondary mass spectrometry detection used the “Top Speed” mode, with 15,000 (at 200 m/z) secondary mass spectrometer resolution, 75% AGC, 22 ms maximum injection time, and 33% peptide fragmentation collision energy. The Orbitrap Exploris™480 mass spectrometer was used to generate raw mass spectrometry detection data. The proteins were annotated using the *Homo sapiens* SP database (protein count: 20,407, database: UniProt, access date: 2023.03.07) and Proteome Discoverer 2.4 software. The processed data underwent further processing, including overall data quality deviation statistics, evaluation and statistical analysis of the identified peptides, and evaluation and statistical analysis of the identified proteins for data quality control. The filtered data were then used for bioinformatics analysis.

### 2.5 Bioinformatics analysis

The differentially expressed proteins were screened based on a fold change (FC) ≥1.5 and a *P*-value ≤0.05. The differentially expressed proteins were plotted using a statistical bar chart. Then, the FC of each differentially expressed protein was log-transformed to base 2, and the absolute *P*-values were log-transformed to base 10; the data were presented as volcano plots. Next, gene ontology (GO) and Kyoto Encyclopedia of Genes and Genomes (KEGG) analyses of the differentially expressed proteins were performed.

### 2.6 Western blot validation of differential protein expression in samples

Total protein was extracted from tophi and joint cavity effusion in the patients with chronic and acute GA, respectively (n = 30 each group), and the protein concentration was measured. Proteins were separated on using the TGX Stain-Free FastCast Acrylamide Kit. After electrophoresis, the gel was exposed to UV light for 1 minute, and images were directly captured using a chemiluminescence imaging system. After imaging, the separated proteins on the gel were transferred to a PVDF membrane using the wet transfer method. The PVDF membrane was placed in 5% non-fat milk TBST and incubated at room temperature on a shaker for 1 hour. The primary antibodies were diluted in a universal antibody diluent. Their dilution ratios were as follows: GPX4(1:1,000), FTH1(1:500), and ALOX15(1:5,000).. The PVDF membrane was incubated overnight at 4 °C with the primary antibodies. After washing the membrane with TBST, it was incubated at room temperature for 1 hour with the respective secondary antibody diluted in the universal antibody dilution buffer (1:5000 for all secondary antibodies). Following the incubation with the secondary antibodies, the membrane was washed with TBS, and the ECL chemiluminescent solution was developed in a fully automated chemiluminescent fluorescence image analysis system under light-protected conditions. The gel and chemiluminescent images were analyzed using grayscale scanning (Image J); the gel images served as the total protein reference to calculate the expression levels of each target protein.

## 3 Statistical analyses

Statistical analysis was performed using SPSS 27.0. Quantitative data met the Shapiro-Wilk normality test and are presented as mean ± standard deviation (M ± SD). Comparisons between groups were assessed using the F test to compare variances to determine homogeneity of variance. If variances were homogeneous, an independent samples t-test was applied; if variances were heterogeneous, Welch’s t-test was used. For non-normally distributed data, the Mann-Whitney U test was employed. A *P* value < 0.05 was considered statistically significant.

## 4 Results

### 4.1 Baseline data

**Figure 2:**
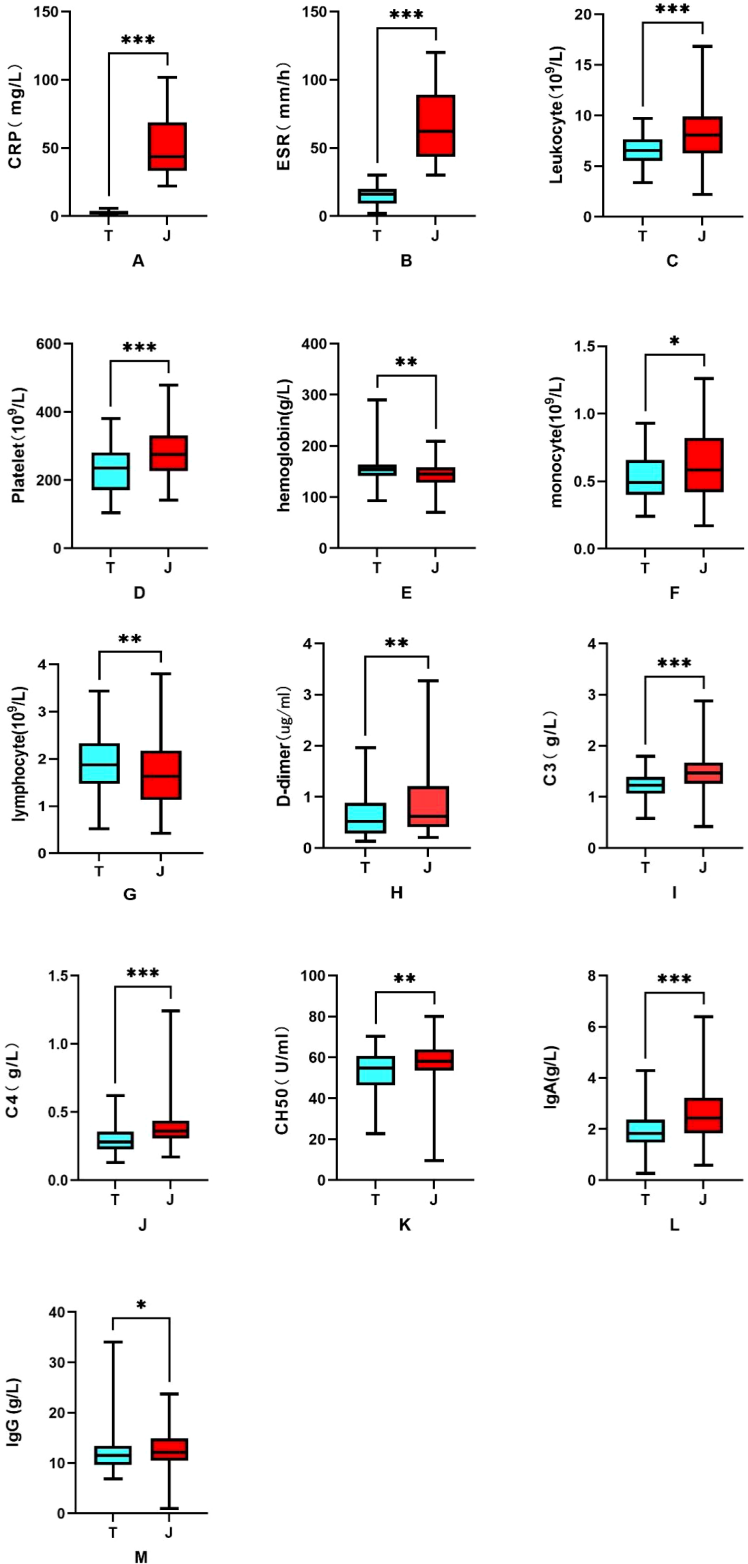
Laboratory parameters of the patients with GA. **(A)** CRP; **(B)** ESR; **(C)** leukocyte; **(D)** PLT; **(E)** HB; **(F)** M; **(G)** LYM; **(H)** D-dimer; **(I)** C3; **(J)** C4; **(K)** CH50; **(L)** IgA and **(M)** IgG. T refers to chronic tophi in GA, and J refers to joint cavity effusion in the acute phase of GA. *P < 0.05; **P < 0.01; ***P < 0.001.

This study included 100 patients with chronic GA complicated by tophi, including 99 males and 1 female. Their ages ranged from 25 to 70 years, with an median age of 51.50 years. The median duration of the disease was 10 years. The median C-reactive protein (CRP) level was 2.65 mg/L, and the median erythrocyte sedimentation rate (ESR) was 16 mm/h. The study also included 100 patients with acute GA complicated by joint cavity effusion, including 93 males and 7 females. Their ages ranged from 27 to 70 years, with an median age of 58 years and an median disease duration of 10 years. Moreover, their median CRP level was 43.50 mg/L, and median ESR was 62 mm/h. The patients with chronic GA complicated by tophi exhibited significantly higher hemoglobin (HB), lymphocyte (LYM) levels as compared to those with acute GA complicated by joint cavity effusion. On the other hand, the patients with acute GA complicated by joint cavity effusion had significantly higher levels of CRP, ESR, leukocyte, platelets (PLT), monocytes (M),D-dimer, C3, C4, CH50, IgA and IgG as compared to those with chronic GA complicated by tophi. The details results are presented in **Table 2**, **Figure 2**.

**Table 2:** Clinical data of GA patients with chronic tophi and acute joint cavity effusion.

Group	Tophi M(P25,P75)	Joint cavity effusion M(P25,P75)	Normal range	Z	*P*-value
Age (years)	51.50(42.50,64)	58(45.68)		-1.4	0.158
Duration of illness (years)	10(7.12)	10(5.15)		-0.307	0.759
SUA (μmol/L)	515(413,517)	453.50(389, 549)	143-339	-2.172	0.3
CRP (mg/L)	2.65(1.50,3.35)	43.50(33.20, 68.78)	<3	-10.45	<0.001***
ESR (mm/h)	16(9,20)	62(44,88)	0-25	-12.19	<0.001***
leukocyte (10^9^/L)	6.54(5.54,7.67)	8.09(6.26,9.08)	3.5-9.5	-4.60	<0.001***
PLT (10^9^/L)	226(180,275)	234.50(170.75,280.50)	125-350	-4.22	<0.001***
HB (g/L)	153.50(141.50, 163.25)	128(105,158)	115-150	-0.28	0.005**
M (10^9^/L)	0.49(0.40, 0.66)	0.59(0.42,0.82)	0.1-0.6	-4.27	0.014*
LYM (10^9^/L)	1.88(1.48,2.34)	1.63(1.14,2.18)	1.1-3.2	-2.81	0.005**
D-dimer (ug/ml)	0.41(0.25,0.62)	0.52(0.28,0.89)	0-0.5	-3.09	0.002**
C3 (g/L)	1.23(1.06,1.39)	1.47(1.26, 1.67)	0.9-1.8	-6.69	<0.001***
C4 (g/L)	0.28(0.23, 0.36)	0.31(0.22, 0.36)	0.1-0.4	-5.26	<0.001***
CH50 (U/ml)	54.85(46.35, 60.78)	58.10(53.65, 63.85)	36-64	-2.85	0.005**
IgA (g/L)	1.83(1.47, 2.36)	2.43(1.84, 3.23)	0.7-4	-4.89	<0.001***
IgG (g/L)	11.50(9.61, 13.39)	12.17(10.49, 14.87)	7-16	-2.561	0.01*

### 4.2 Proteomics and bioinformatics analyses

#### 4.2.1 Protein quality assessment results

**Figure 3:**
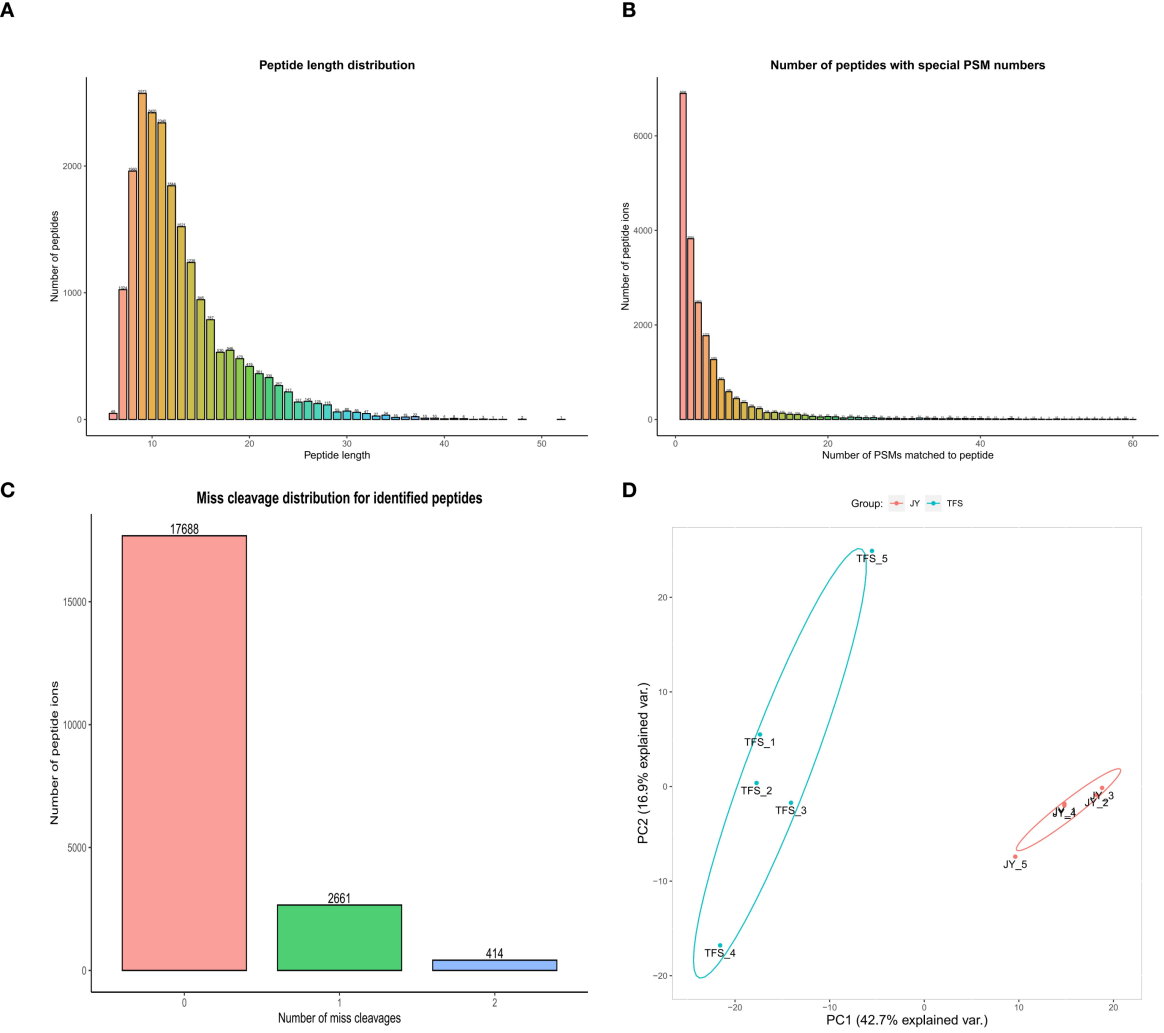
Protein quality assessment results. **(A)** Peptide length. The horizontal axis represents the distribution of peptide segment lengths, and the vertical axis represents the number of peptide segments under different length distributions. Generally, the suitable peptide segment length distribution detectable by mass spectrometry is in the range of approximately 7–40 amino acids. **(B)** Number of PSMs matched to peptide. The x-axis shows the number of spectrum matches, and the y-axis shows the number of peptides under different PSM distributions. The higher the number of PSM matches for a peptide, the higher the reliability of peptide identification. **(C)** Number of miss cleavages. The x-axis shows the number of missed cleavage sites, and the y-axis shows the number of peptides under different missed cleavage conditions. This result reflects the efficiency of enzymatic digestion; a higher proportion of no missed sites indicates better enzymatic digestion efficiency. **(D)** Principal component analysis. An intuitive analysis of intergroup differences and intragroup reproducibility, helping to identify outlier data. Samples of the same color form a group; the more clustered the groups are, the better the intragroup reproducibility; the more distinct the separation between groups, the more significant the intergroup differences. Points represent samples, and circles represent confidence intervals. TFS: chronic phase (with tophi); JY: acute phase (with joint cavity effusion).

The analysis of peptide segment length (**Figure 3A**) showed that most peptide segments contained 7–20 amino acids; this was consistent with the general pattern based on enzymatic digestion and MS fragmentation methods. The peptide segment lengths identified using MS also met quality control requirements. The statistical distribution of the number of PSMs identified per peptide segment and the distribution of missed cleavage sites (**Figures 3B, C**) showed that a high proportion of peptide segments had zero missed cleavage sites (85.2%), meeting quality control requirements. The more PSMs matched to a peptide segment, the higher the reliability of the peptide segment identification. The more peptide segments with zero missed cleavage sites, the more thorough the enzymatic digestion, and the more reliable the identification results. Principal component analysis (PCA, **Figure 3D**) showed significant protein differences between the groups and strong correlations within groups, thereby meeting the MS standards.

#### 4.2.2 Results of the differential protein analysis

**Figure 4:**
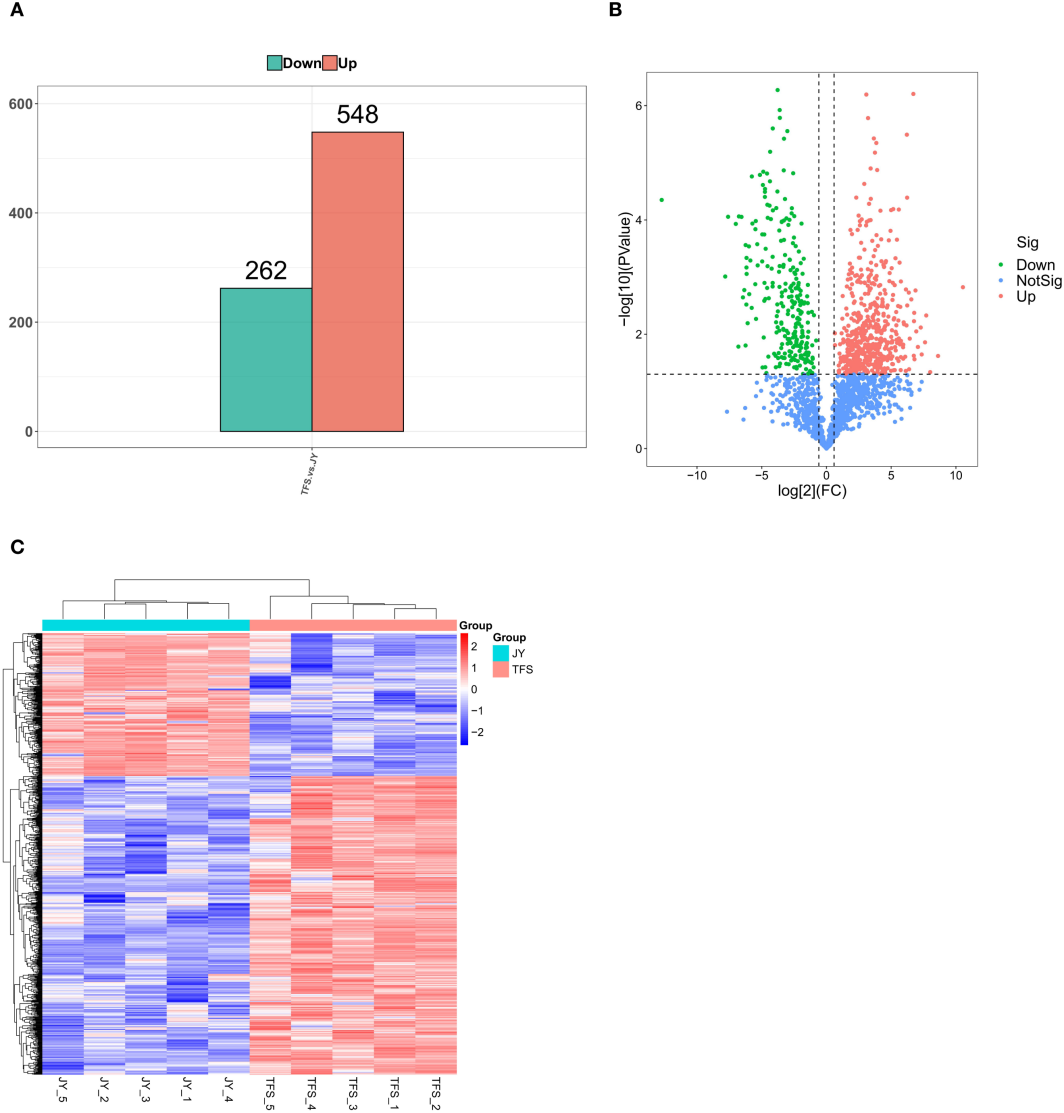
Differential protein analyses. **(A)** Differential protein screening results, showing the statistical analysis of the number of differentially expressed proteins in different comparison groups. Red and green represent the number of upregulated and downregulated differentially expressed proteins, respectively. **(B)** A volcano plot reflecting the overall differences in protein expression. The x-axis is represented by log2 (FC), and the y-axis is represented by -log10 (*P*-value). Each point represents a protein, and colors are used to distinguish whether a protein is differentially expressed. The two dashed lines on the x-axis represent the FC threshold values, with the left and right lines corresponding to FC <1/1.5 and FC >1.5, respectively. The dashed line on the y-axis represents the *P*-value threshold, with points above the line indicating *P*-value <0.05 data. Combining the FC and *P*-value metrics, red points in the upper right corner and green points in the upper left corner represent upregulated and downregulated differentially expressed proteins, respectively. **(C)** Heatmap of the differentially expressed proteins. The horizontal axis represents each sample, and the vertical axis represents the differential proteins. The colors in the figure indicate protein expression levels, and the red and blue color intensities indicate higher and lower expression levels, respectively. TFS: chronic phase (with tophi); JY: acute phase (with joint cavity effusion).

The results showed that a total of 810 differentially expressed proteins were obtained from the two groups, including 548 upregulated proteins and 262 downregulated proteins (**Figure 4**).

#### 4.2.3 GO and KEGG analyses of the differentially expressed proteins

**Figure 5:**
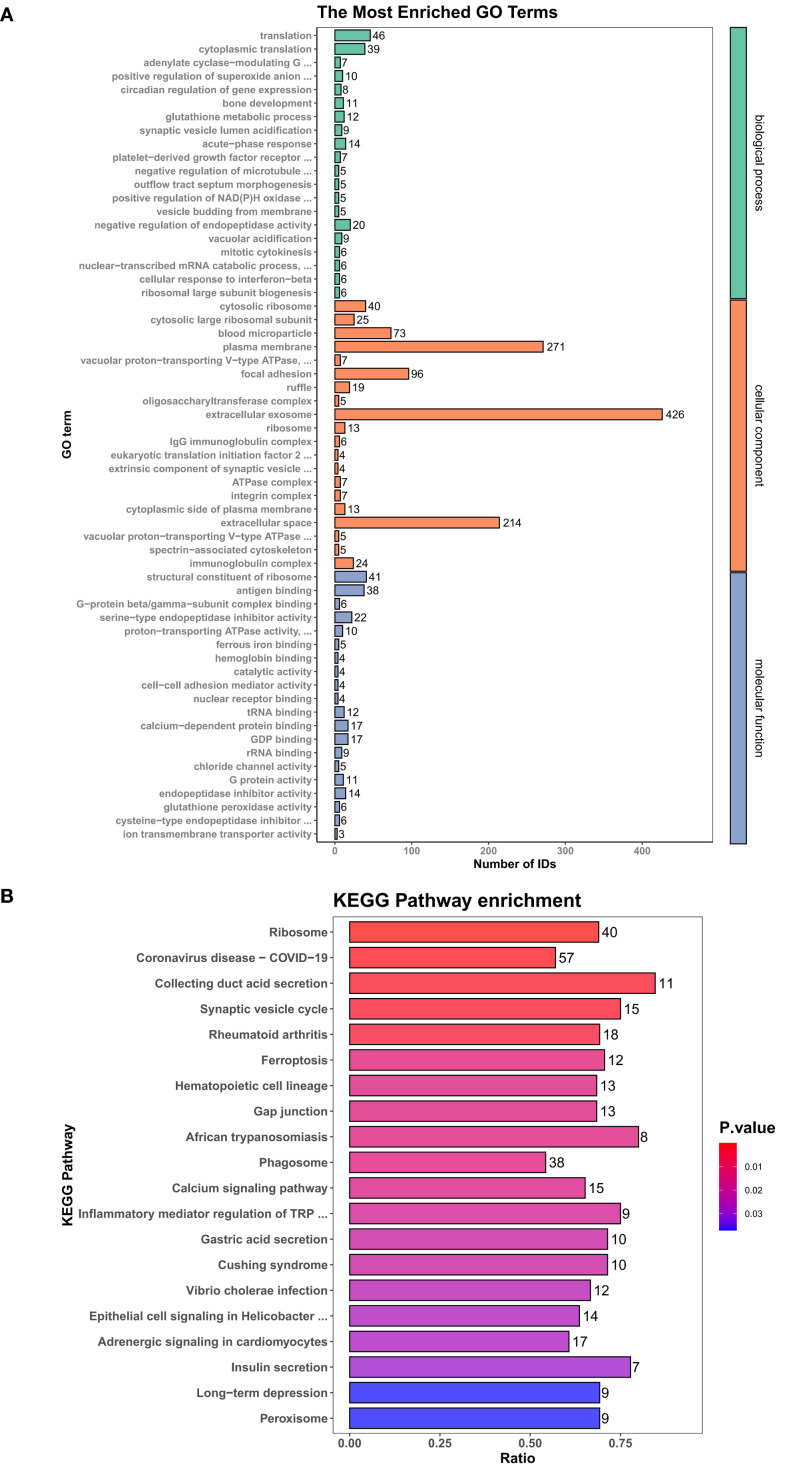
GO analysis and KEGG enrichment analysis results of the differentially expressed proteins. **(A)** GO enrichment triple column chart. The horizontal axis represents the number of proteins identified for each GO term, and the vertical axis represents the three subcategories of GO (BP, biological process; CC, cellular components; and MF, molecular functions) with different colored bars. If there were fewer than 20, all are displayed. **(B)** KEGG enrichment bar chart. The Y-axis represents the top 20 terms in the enrichment table, and the X-axis displays the enrichment ratio values. The bar chart colors represent the enrichment *P*-values, and the numbers on the right represent the number of enriched proteins.

**Figure 5A** shows a bar chart of the top 20 most enriched GO terms. The enriched biological processes mainly included translation, cytoplasmic translation, negative regulation of endopeptidase activity, and acute-phase response. The enriched cellular components mainly included extracellular exosomes, plasma membrane, extracellular space, focal adhesion, and blood microparticles. Moreover, the enriched molecular functions mainly included structural constituent of ribosome, antigen binding, serine-type endopeptidase inhibitor activity, calcium-dependent protein binding, and GDP binding, among others. The top 20 enriched KEGG pathways are shown in a bar chart (**Figure 5B**), which mainly included ribosome, coronavirus disease - COVID-19, phagosome, rheumatoid arthritis, adrenergic signaling in cardiomyocytes, calcium signaling pathway, epithelial cell signaling in *Helicobacter*, hematopoietic cell lineage, gap junction, and ferroptosis. A previous study showed that iron overload may be involved in the pathogenesis of the acute phase of GA (12). Animal meat and seafood rich in purines are more likely to trigger acute GA as compared to vegetables rich in purines. This may be related to the fact that animal-based foods are also rich in iron, which is more easily absorbed and utilized by the human body (13). Thus, the differentially expressed proteins and their GO/KEGG enrichment analyses identified three proteins related to ferroptosis as validation targets: GPX4, FTH1 and ALOX15.

#### 4.2.4 Validation of the differentially expressed proteins

**Figure 6:**
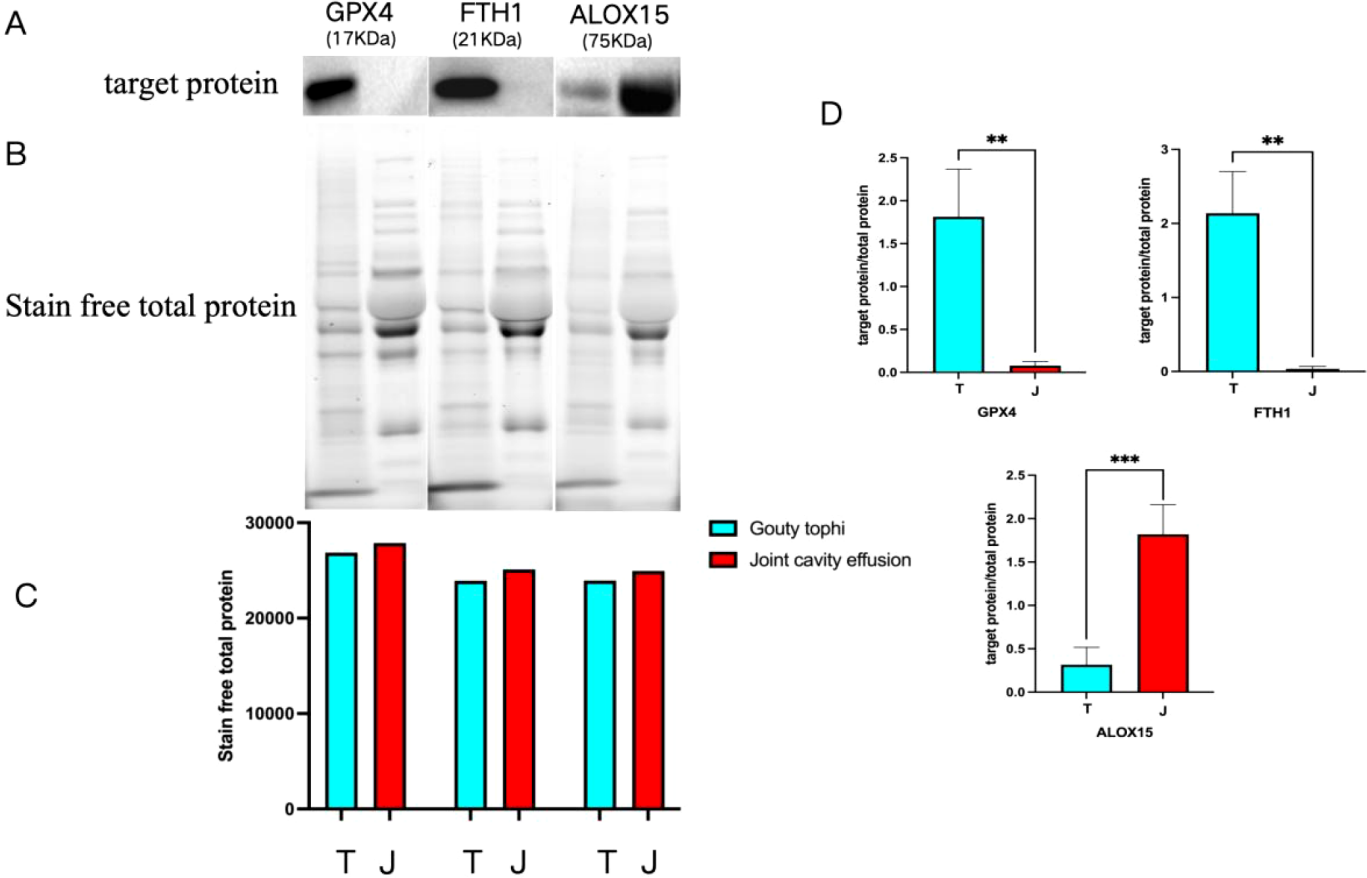
Results of the differential protein validation experiment. **(A)** Target protein band. **(B)** Stain-Free total protein band. **(C)** Total protein bar chart (6). Target protein/total protein bar chart. **P < 0.01; ***P < 0.001. T: chronic phase (with tophi); J: acute phase (with joint cavity effusion).

GA patients in the chronic phase showed increased expression of GPX4 and FTH1 (*P* < 0.01); GA patients in the acute phase showed increased expression of ALOX15(*P* < 0.001). Among these, the gray values of the target proteins (**Figure 6A**) were divided by those of their corresponding total proteins (**Figure 6B**) (14), and the bar charts were plotted to obtain *P*-values (**Figure 6D**). The bar charts of total proteins corresponding to each target protein are shown in **Figure 6C**. The differences in total protein between tophi and joint effusion were within the acceptable error range and considered reliable protein quantification data.

## 5 Discussion

### 5.1 Discussion of experimental methodology

In validation experiments, joint cavity effusion and tophi showed complex and distinct protein compositions. Therefore, the selection of internal controls was crucial for ensuring the accuracy of experimental results and facilitating the exploration of molecular mechanisms. At first, this study attempted to use GAPDH, β-actin, and albumin (ALB) as internal controls; however, the two groups belonged to different types of proteins, and their expressions were unstable. The literature review suggested that the stain-free method is a more stable and precise internal reference for the quantitative analysis compared to the commonly used housekeeping proteins (actin, tubulin, and GAPDH). Moreover, the stain-free method also has a more ideal linear dynamic range, making it a suitable alternative to traditional housekeeping internal protein references. The stain-free method enables the visualization of total proteins on gels without the need for additional labeling or antibodies, providing accurate and reliable protein quantification data. This method not only simplifies experimental procedures but also significantly improves the accuracy and reliability of experimental results (15, 16). It is a simple method for the semi-quantification of protein levels in different samples and can detect total protein levels within a wide linear dynamic range (14). Therefore, this study ultimately selected the stain-free total protein technology.

### 5.2 Proteomics testing and discussion of baseline data

The baseline data collected from 100 patients in each group showed that the levels of complement C3, C4, CH50, IgA and IgG were significantly higher in the patients with acute GA compared to those with chronic GA. Studies have shown that C3 mediates various pathological pains, including postoperative pain, inflammatory pain, neuropathic pain, complex regional pain syndrome, and arthritis pain, among others (17). An abnormal elevation of complement C3 and C4 serum levels can induce immune cells to produce important pro-inflammatory cytokines, such as interleukin-1β (IL-1β), IL-6, and tumor necrosis factor-α (TNF-α) (18). CH50, similar to CRP, is highly sensitive to inflammation (19). IgA is an effective stimulant for activating neutrophils after cross-linking of CD89 on the neutrophil surface (20, 21)and is associated with inflammation. IgG induces a hyperinflammatory state in macrophages (22). In the current study, the patients’ complement C3, C4, CH50, IgA and IgG levels were positively correlated with the inflammatory state of their condition. Therefore, the baseline data from the current study aligned with the current inflammation-related research. Moreover, proteomics analysis revealed an enrichment of blood microparticles in tophi. Studies have indicated that blood microparticles promote and support blood coagulation (23). Therefore, targeting therapy for coagulation abnormalities may become a novel therapeutic strategy for treating platelet deposits.

### 5.3 Discussion of verification experiment results

In this study, the quantitative proteomics analysis of tophi from chronic-phase GA patients and joint cavity effusion from acute-phase GA patients identified 810 differentially expressed proteins, including 548 upregulated and 262 downregulated proteins. The GO and KEGG pathway enrichment analysis of the differentially expressed proteins narrowed down this list to three proteins associated with ferroptosis: GPX4, FTH1 and ALOX15. Western blot analysis of the tophi from chronic-phase GA patients and joint cavity effusion from acute-phase GA patients confirmed the differential expression of these three proteins. The results showed that the expression of GPX4 and FTH1 significantly increased in chronic-phase GA patients (*P* < 0.01), while ALOX15 expression increased in patients with acute GA(*P* < 0.001).

Cell death is an extremely complex process, including energy-dependent programmed cell death and energy-independent cell necrosis (24). Ferroptosis differs from other forms of cell death, such as apoptosis and autophagy. It was first proposed by Stockwell et al. (25, 26) in 2012. Its occurrence is primarily associated with iron metabolism, lipid peroxidation (LPO), and amino acid metabolism. Morphologically, ferroptosis is characterized by a reduction in mitochondrial cristae and rupture of the mitochondrial outer membrane, leading to mitochondrial dysfunction (25, 27). Unlike other forms of cell death, it spreads rapidly through cell populations in a wave-like manner (28) and triggers a strong immune response. Ferroptosis can trigger the body’s immune mechanisms, leading to the release of pro-inflammatory cytokines, such as IL-1β, TNF-α, and IL-6, thereby promoting the expansion of inflammatory responses (29).

GPX4 is one of the most important inhibitory molecules in the ferroptosis process. In the presence of glutathione (GSH), GPX4 acts as a “peroxidase-inhibiting protein”on liposomes and biological membranes and is capable of reducing various oxidative substrates, thereby preventing LPO and protecting the integrity of cell membranes (30). The Xc-/GSH/GPX4 axis is a central regulatory pathway in ferroptosis. Most iron-inducing agents, such as Erastin (31) and GPX4 inhibitors (32), can hinder the antioxidant activity of this axis. As a key downstream antioxidant enzyme of the Xc-/GSH/GPX4 axis, the absence or dysfunction of GPX4 can cause the accumulation of intracellular peroxides, triggering ferroptosis (33). Studies have shown that GPX4 can regulate the Nuclear Factor kappa-light-chain-enhancer of activated B cells (NF-κB) pathway, reduce reactive oxygen species (ROS) levels, and inhibit the production of inflammatory factors (34).

FTH1 is one of the main components of serum ferritin. Ferritin is a cytoplasmic iron storage complex protein that can chelate up to 4,500 iron atoms. Ferritin is an important antioxidant molecule in the cells and stores iron (35). It is the primary regulator of iron oxidase activity, oxidizing Fe²^+^ to Fe³^+^ and storing excess cellular iron, thereby reducing the production of ROS and tissue damage (36). In addition to maintaining iron homeostasis, ferritin can also function as an oxidative stress regulator and inhibitor of cell apoptosis (37, 38).

ALOX15 is a non-heme dioxygenase and a subtype of lipoxygenases (LOXs). It can catalyze the free and accelerated oxidation of polyunsaturated fatty acids (PUFAs), producing a series of bioactive lipid products that are involved in human inflammatory responses and immune regulation (39). The intracellular iron accumulation and LPO are two central events in ferroptosis. Arachidonic acid (AA) is a PUFA essential for human health. In cell membrane structures, PUFAs are more susceptible to being attacked by oxygen free radicals due to the instability of their double bond structure, leading to peroxidation. Under the catalysis of LOXs, the production of lipid peroxides induces ferroptosis (40). ALOX15 can catalyze the metabolism of AA, producing lipid peroxides that affect intracellular protein activity and plasma membrane stability, thereby promoting the occurrence of ferroptosis (41).

In this study, the two ferroptosis-inhibiting factors, including GPX4 and FTH1, were significantly elevated in the chronic tophi, while the expression of the ferroptosis-promoting factor ALOX15 in acute joint cavity effusion was significantly higher than in chronic tophi. Therefore, it was speculated that ferroptosis is in an active state during the acute phase of GA, leading to a strong immune response and promoting the occurrence and development of inflammation.

## 6 Outlook

Using proteomics analysis, validation experiments, and stain-free total protein technology, this study provided a reference for future studies on the local pathological mechanisms of GA and offered predictive directions for its pathological changes. The current study innovatively explored potential ferroptosis-related predictive factors and therapeutic targets during the acute and chronic phases of GA. Taking the local inflammation and clinical manifestations of GA patients as the key points for current treatment, tophi and joint cavity effusion were selected as targets, representing an innovation in both clinical and basic experiments. The results identified key proteins in the acute phase of GA that are closely associated with ferroptosis. The ferroptosis-promoting proteins, such as ALOX15, showed increased expression in the acute phase of GA. In contrast, the ferroptosis-inhibiting factors, such as GPX4 and FTH1, increased in the chronic phase of GA. Therefore, it was speculated that ferroptosis-inhibiting factors might participate in the chronic progression of GA by limiting the toxicity of free iron.

There were certain limitations to this study. The iron levels are not a standard test item yet, and due to cost constraints, the iron levels in the patients in this study were not measured. The future studies should include iron levels as a corresponding test item. Due to time and cost constraints, the number of clinical samples collected from the patients in both groups was limited; therefore, further validation with a larger sample size is needed. Future studies should focus on exploring the mechanism linking the pathogenesis of acute GA to ferroptosis in animal and cell models to fully validate these predictions; this will also be a future direction for our research. This study focused on localized research, and future efforts should aim to conduct a comprehensive, systematic investigation into the mechanism of ferroptosis in GA.

## Data Availability

The mass spectrometry proteomics data have been deposited to the ProteomeXchange Consortium (https://proteomecentral.proteomexchange.org) via the iProX partner repository with the dataset identifier PXD075785.
